# Tryptophan as a biomarker of pregnancy-related immune expression and modulation: an integrative review

**DOI:** 10.3389/frph.2024.1453714

**Published:** 2025-01-23

**Authors:** Stephanie Prescott, Natasa Billeci, Melissa Gotcher, Sapna Patel, Addison Almon, Hailey Morgan, Danielle Abukhalaf, Maureen Groer

**Affiliations:** ^1^ College of Nursing, University of South Florida, Tampa, FL, United States; ^2^ School of Nursing, Inova Health Services, Fairfax, VA, United States; ^3^ College of Nursing, University of Tennessee, Knoxville, TN, United States

**Keywords:** biomarkers, fetal outcomes, pregnancy outcomes, tryptophan, tryptophan metabolism

## Abstract

**Background:**

Degradation pathways of Tryptophan (TRP) are implicated in a spectrum of physiological adaptations and outcomes associated with pregnancy. The immunomodulatory role of TRP and its metabolites through the indoleamine 2,3-dioxygenase (IDO) pathway is particularly relevant to pregnancy due to its potential influence on maternal and fetal immune tolerance and the mother's health.

**Methods:**

A targeted literature search was conducted via PubMed, Web of Science, and Embase, focusing on maternal serum TRP levels in pregnancy. We included original human subject research on maternal serum TRP, published in English within the last five years. We included 16 quality studies with direct measurement of TRP in pregnancy including ten prospective cohorts, four case-control studies, and two cross-sectional studies.

**Results:**

TRP levels are reduced both pre- and postnatally in women with depressive symptoms, but not during pregnancy, though the TRP/Kynurenine pathway is disturbed during pregnancy in women with depressive symptoms, women with prolonged labor, women with gestational hypertension, and in adverse outcomes of pregnancy including prematurity and growth restriction.

**Conclusion:**

TRP and its metabolites hold promise as biomarkers for various pregnancy-related outcomes. Future research should aim to clarify the mechanisms by which TRP metabolism influences maternal and fetal health outcomes.

## 1 Introduction

Tryptophan (TRP) is an essential amino acid that plays a role in a multitude of biological processes. Obtained primarily from the diet, TRP is required for protein production and immune regulation. Normal plasma levels of TRP are 70 ± 10 µmol/L for males and 65 ± 10 µmol/L for females (1). TRP is heavily regulated in the body, as both a surplus and a lack of the amino acid can disrupt homeostasis and normal biological processes. Factors influencing TRP catabolism include inflammation, body mass index (BMI), diet, and mood disorders (2). The body can utilize TRP via two metabolic pathways: the kynurenine (KYN) pathway and the serotonin pathway (3). The KYN pathway is the most prominent of the two metabolic pathways, involving the catabolism of TRP into KYN, which converts over 95% of TRP in the body (4). TRP is converted to KYN by the enzymes indoleamine 2,3- dioxygenase (IDO1), indoleamine 2, 3-dioxygenase-2 (IDO2), and tryptophan 2,3 –dioxygenase (TDO) (5). Along the KYN pathway, TDO converts TRP into KYN in the liver, while IDO1 and 2 convert TRP to KYN in neuronal cells, microbes, and the liver (2). The serotonin pathway of TRP metabolism results in TRP conversion into L-5-hydroxytryptophan (5-HTP) by tryptophan hydroxylase in enterochromaffin cells or by tryptophan hydroxylase 2 in enteric or central neurons. Serotonin can also then be converted into melatonin in the pineal gland. TRP is the sole substrate for synthesizing serotonin, a neurotransmitter that plays a role in mood regulation, and melatonin, a neurotransmitter that helps regulate sleep cycles (6).

Pathways of TRP metabolism can easily be activated or altered by stress and pro-inflammatory conditions (7). The TDO enzyme is activated by stress (cortisol) and regulates the majority of KYN metabolites produced by the KYN pathway. During inflammation, however, the IDO enzymes assume a more prominent role in TRP metabolism due to their role in immune modulation and activation by proinflammatory cytokines such as interferon gamma (IFN-γ). One method of measuring TRP metabolism via the IDO/TDO pathways is measuring the kynurenine to tryptophan ratio (KTR), which may change in response to IDO and TDO enzyme activity (5). During pregnancy-associated physiologic inflammation and stress, TRP catabolism shifts mainly to the IDO pathway (2).

**Figure 1:**
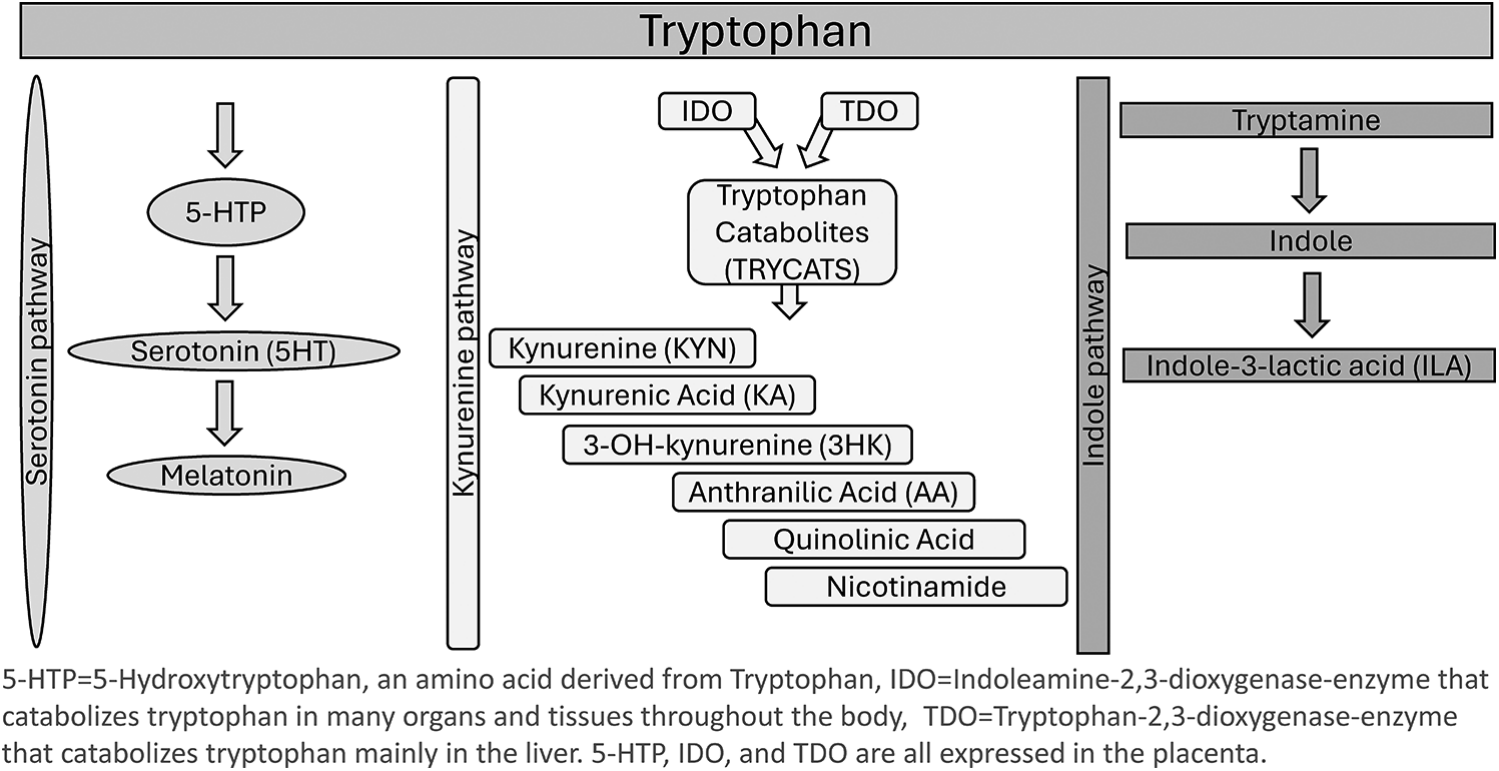
Tryptophan metabolic pathways.

The interplay between TRP metabolism and pregnancy outcomes highlights the connections between maternal nutrition, immune response, and psychological health. During pregnancy, significant metabolic changes occur to support the mother and developing fetus. One such change involves TRP, an essential amino acid with roles in protein synthesis, serotonin production, and immune function. Pregnancy increases metabolic demands and raises the potential for oxidative stress and inflammation. The placenta expresses indoleamine 2,3-dioxygenase 1, an enzyme that breaks down TRP, leading to decreased TRP levels in the mother's blood. This reduction in TRP is thought to modulate immune tolerance during pregnancy, as lower TRP levels can reduce T-cell proliferation, helping to prevent the maternal immune system from attacking the fetus (4). See Figure 1 for an illustration of TRP metabolism and pathways.

There is no comprehensive synthesis of data in current scientific literature regarding the impact of TRP and its metabolites during pregnancy on the mother and the fetus. The Badawy hypothesis suggests that altered TRP metabolism during pregnancy may contribute to mood disorders by affecting serotonin synthesis, highlighting the complex interplay between TRP levels, placental function, and maternal health. Badawy also suggests an alternative interpretation of the mechanisms of the decrease in maternal circulating TRP involving modulation of TRP disposition, but not IDO induction (8). Fluctuations in TRP levels have been associated with various perinatal complications, suggesting its role extends beyond basic nutritional requirements to influence broader physiological and developmental processes (2). As such, deciphering the nuanced effects of TRP during pregnancy could offer valuable insights into preventive care and therapeutic strategies, enhancing both maternal and neonatal well-being. For this review we sought to synthesize current knowledge on the impact of TRP and its metabolites on pregnancy-related outcomes while considering how metabolic pathways might be leveraged to optimize perinatal care and guide clinical practices.

## 2 Methods

### 2.1 Search strategies

A literature search was conducted on December 28, 2023, using PubMed, Web of Science, and Embase electronic databases. Search terms included “tryptophan,” “pregnancy”, “maternal serum”, and “maternal blood”. Aggregated results from the initial search of each database were uploaded to Rayyan.ai (9) for duplicate identification and removal, with the remaining articles being assessed for relevance and compliance with the inclusion and exclusion criteria.

### 2.2 Inclusion & exclusion criteria

We included studies that conducted original research on human subjects involving peripheral blood draws to measure maternal serum TRP levels and were published in English within the past five years. We excluded articles focusing on TRP in non-pregnant populations, those lacking TRP measurements during pregnancy, studies measuring TRP solely in fetal, placental, or cord blood samples, and those involving control groups with pre-gestational illnesses, but no healthy controls. Studies concentrating on dietary TRP intake and those of poor quality were also not considered for the review.

### 2.3 Search outcome

**Figure 2:**
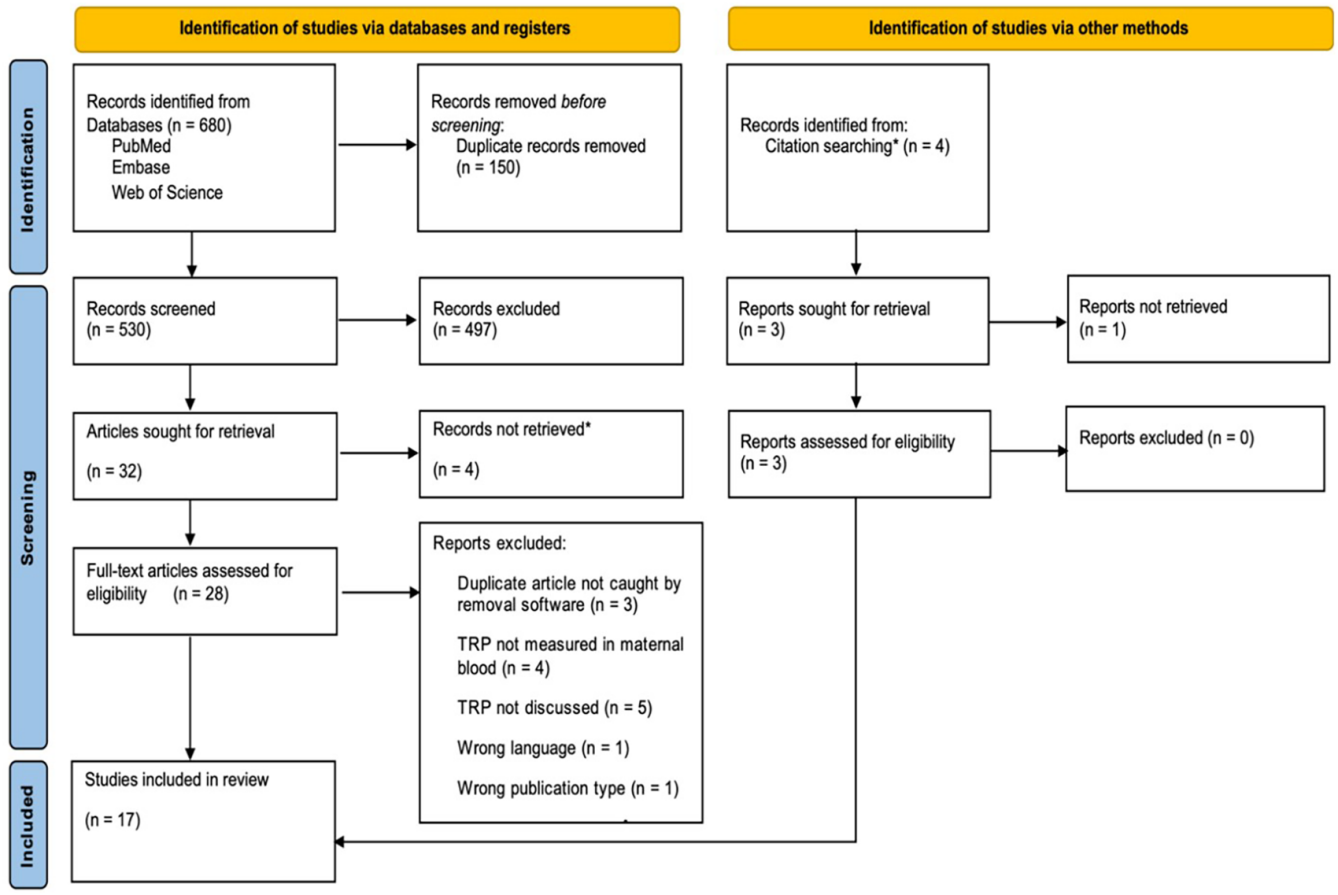
PRISMA flow chart based on PRISMA-systematic review guidelines. *The four reports not retrieved were poster presentations; HM searched for associated journal article publications and included them for eligibility review if found. Source (10): Adapted from The PRISMA 2020 statement: An updated guideline for reporting systematic reviews by Page et al. (10) (http://doi.org/10.31222/osf.io/v7gm2).

A search across PubMed, Web of Science, and Embase yielded 680 studies, reduced to 530 after removing 150 duplicates. Screening these, we discarded 497 and could not retrieve four as they were solely abstracts for poster presentation, leaving 28 for a full review. After excluding three unrecognized duplicates and 11 that did not meet our criteria, and adding three relevant studies found through citations, we finalized 16 studies for the review. These included 10 prospective cohorts, four case-control studies, and two cross-sectional studies. See Figure 2.

### 2.4 Data evaluation

Articles were evaluated using a rating system. According to the study design, the National Institutes of Health (NIH) quality assessment tools (https://www.nhlbi.nih.gov/health-topics/study-quality-assessment-tools, July 2021) were utilized to assign scores corresponding to good, fair, or poor ratings. A summary of review findings and article ratings can be found in Table 1.

**Table 1:** Summary of selected manuscripts.

Citation	Purpose	Sample size	Research design	Variables/instruments	Selected findings
Teshigawara et al. (7)	Plasma TRP and metabolites in pregnant women with depressive symptoms	non-depressive (ND) (*n* = 62), postpartum depressive (PD) (*n* = 15), temporary gestational depressive (TG) (*n* = 22), and continuous depressive (CD) group (*n* = 33)	Longitudinal	Edinburgh Postnatal Depression Scale (EPDS). TRP, KYN, KA, AA, and 3HAA. Salivary cortisol, IL-6	KYN, KYNA higher and 3HAA lower in PD. In the ND group, postpartum levels of TRP, KYN, 3HK, and KA were significantly higher than during pregnancy. There was no significant difference in plasma levels of TRP of its metabolites between the TG group or the CD group and the ND group. The ratio of plasma KYN level during postpartum to that during pregnancy in the PD group was significantly lower than in the ND group (*p* < 0.01). In the ND group, the KYN/TRP ratio during the postpartum period was higher, but 3HK/KYN, AA/KYN and 3HAA/3HK ratios during the postpartum period were significantly lower than those during pregnancy. KYN/TRP and KA/KYN ratio during the pregnancy period in the PD group were higher than those in the ND group.
Nazzari et al. (11)	TRP and its metabolites in pregnant women with depressive and anxiety during pregnancy and postpartum.	*N* = 110	Longitudinal	Edinburgh Postnatal Depression Scale (EPDS) and State-Trait Anxiety Inventory subscale (STAI-S), Cortisol, IL6, TRP, KYN, KTR	EPDS Scores equal or above cutoff: 17% in pregnancy and 2 days after delivery, about 10% 3 and 12 months postpartum. STAI Scores equal or above cutoff: 24% during pregnancy, 25% two days after delivery, 31% after three months, and 30% after 12 months postpartum. Mean salivary Cortisol levels(µg/dl) at wake were 0.38 (n-109), at 30 min were 0.50 (*n* = 108), and at bedtime were 0.18 (*n* = 110). Mean IL-6 (pg/ml) levels were 1.68 (*n* = 97). Mean Trp levels were 11.12 (*n* = 97). Mean Kyn (ng/ml) levels were 207.60 (*n* = 97). Mean Kyn/Trp*1,000 ratio was 19.85 (*n* = 95).
Zhao et al. (12)	tryptophan metabolites in maternal and umbilical blood samples in normotensive and preeclamptic pregnancies.	normotensive (NT) pregnancies (*n* = 20 with 10 female and 10 male fetuses) and preeclamptic (PE) pregnancies (*n* = 20 with 10 female and 10 male fetuses). l 40 women singleton pregnancies.	Cross-sectional	25 tryptophan metabolites in serum were measured using liquid chromatography with tandem mass spectrometry. The effects of Kyn and ILA on human umbilical vein epithelial cells (HUVEC) were also measured. Cell viability was also assayed. Finally, cell monolayer integrity was examined using an electric cell-substrate impedance sensing (ECIS) system.	Pre-gestational and pregnancy BMI were slightly but significantly (*p* < 0.05) higher in the PE vs. NT group, while gestational ages were lower (*p* < 0.05) in the PE vs. NT group. PE vs. NT groups had lower Fetal birth weights (*p* < 0.05). The concentrations of 9 tryptophan metabolites (Trp, NFK, Kyn, 3-HK, 3-HAA, QA, CVI, ILA, and IAA) were higher (*p* < 0.05) in umbilical than maternal sera. In contrast, the concentrations of L-5-HTP in NT and 5-HT in both NT and PE were higher (*p* < 0.05) in maternal than in umbilical sera. The concentration of ILA was significantly (*p* < 0.05)elevated in PE-maternal (35% above NT) and PE-umbilical(31% above NT) sera. KA (*r* = 0.684, *p* < 0.025) and PA (*r* = 0.641, *p* < 0.031) were positively correlated with proteinuria. Kyn decreased (*p* < 0.05) cell viability by about 19 and 18% at 5 and 10 microM, respectively. Both Kyn and ILA-dose and time-dependently increased (*p* < 0.05) the electrical resistance of the cell monolayer.
Carlson et al. (13)	metabolic pathways in the serum of African American women during late pregnancy that predicted term labor dystocia.	The subjects (*n* = 97) term labor dystocia (*n* = 48), normal labor progression controls (*n* = 49)	Matched Case-control study.	serum samples were analyzed using ultra-high-resolution metabolomics	Metabolic pathways enriched in cases of labor dystocia. Labor dystocia differed from normal controls in androgen and estrogen biosynthesis pathway metabolites (total sample, *p* = 0.028, overlap size 6 of 45; nonobese women, *p* = 0.036, C21-steroid pathways findings and glycosphingolipid pathway metabolites were overrepresented in the labor dystocia group. Maternal BMI and age tryptophan metabolism significantly differentiated labor dystocia cases and controls across all women (*p* = 0.021).
Silvano et al. (5)	Investigate the co-expression of IDO, TDO, and Angiotensin (1–7) in the placenta as well as determine the correlation between plasma levels of Trp and its catabolite Kyn and placental IDO/TDO expression.	*N* = 20	Prospective observational study	RNA extractions and quantification by Real-time PCR for TDO placental expression were conducted to investigate TDO mRNA (TDO2) expression, plasma levels of tryptophan and kynurenine by ELISA	TDO2 placental expression was present in all samples (*n* = 20) on both the maternal and fetal sides of the placenta. No significant difference was present in the percentage of cells expressing IDO1, TDO, or both between the maternal, fetal, or villi surfaces. The ELISA assay revealed mean Kynurenine levels of 597.2 ng/ml mean Trp levels of 8.25 ug/ml in maternal plasma, and the Kyn/Trp ratio was 0.08 + 0.01. This was not significantly correlated with the expression of IDO1 and TDO in the placenta.
Duan et al. (14)	Investigate the association of IDO genetic polymorphisms with postpartum depressive symptoms (PDS).	*n* = 725 women who underwent c-section *n* = 48 with PDS and *n* = 48 without PDS, *n* = 50 carrying the IDO rs10108662 AA genotype and *n* = 50 carrying the IDO rs10108662 AC + CC	Prospective cohort study	Edinburgh Postnatal Depression Scale (EPDS) perinatal serum concentrations of TRP & kynurenine, as well as polymorphisms of the IDO gene. TRP, KYN, and KYN/TRP ratio levels.	variations of IDO1 gene rs10108662 were significantly related to PDS incidence (*p* < 0.05). IDO activity significantly different between the IDO rs10108662 CA + AA, vs. CC, genotypes. A significant change in tryptophan (*p* < .05) and kynurenine (*p* < .05) on postpartum days 1 & 3 compared to the end of term (before c-section). No differences in TRP levels were observed for parturient with PDS, vs. parturient without PDS, at the end of term and postpartum day 1.TRP levels in PDS, vs. non-PDS, were lower on postpartum day 3 (3.69 ± 0.80 g/ml vs. 5.71 ± 1.04 g/ml, *p* < 0.001). KYN levels in PDS, vs. non-PDS, were higher at the end of the term (0.18 ± 0.05 g/ml vs. 0.14 ± 0.04 g/ml, *p* = 0.045, KTR ratios in PDS vs. non-PDS were significantly higher at these same time points
Sha et al. (15)	pro-inflammatory cytokines and neuroactive kynurenine metabolites linked to severity of depressive symptoms.	*n* = 114	Longitudinal	Blood sample, inflammatory cytokines and KYN	Inflammatory cytokines and kynurenine metabolites are associated with depression severity during pregnancy, and postpartum.
Kimmel et al. (16)	tryptophan related metabolites and bile acids in a sample of pregnant women with depressive and anxiety disorders.	*n* = 30	Cross-sectional	Maternal blood and fecal matter collected.	KA KYN from second trimester to third trimester were strongly associated with TUDCA, GCA, TCDCA, and TCA. (2) Secondary bile acid UDCA and its conjugated forms were associated with lower bacterial diversity and positively associated with levels of Lachnospiraceae, a taxa known to produce Short Chain Fatty Acids. (3) History of anxiety associated with UDCA levels.
Keane et al. (17)	Investigate whether GI permeability and systemic inflammation are elevated in mothers who experience higher levels of stress, anxiety, and depression during pregnancy and whether this stress signature is potentially augmented in women with IBS.	Irish Women with IBS (*n* = 105) and no IBS (*n* = 104).	Prospective Case-control	Psych (PSS, EDPS), gut permeability (circulating IFABP and sCD14, LBP, and anti-endotoxin core antibodies), proinflammatory cytokines, CRP, tryptophan, and kynurenine.	positive associations with STAI scores for KTR ratio at 15 weeks’ gestation, IL-6 and covariate-adjusted IP-10 and Trp/Kyn ratio at 20 weeks’ gestation. IBS (maternal anxiety), Trp levels significantly decreased in the high-scoring group compared with moderate- (–707.88 ± 289.38 ng/ml, *p* = 0.044) and low-scoring groups (–1111.14 ± 380.08 ng/ml, *p* = 0.013). (Maternal depression, EDPS):, Kyn concentrations significantly higher in the in the low-scoring group compared with moderate- (34.2 ± 12.13 ng/ml, *p* = 0.017) and high-scoring groups (40.89 ± 14.13 ng/ml, *p* = 0.014).
Roomruangwong et al. (18)	Delineate the associations between the TRYCAT pathway and PMS and perinatal depressive and physio-somatic symptoms.	*n* = 126 third-trimester pregnant depressed and not-depressed groups *n* = 24 age-matched non-pregnant healthy women.	Cross-sectional	Peripheral blood sample Edinburgh Postnatal Depression Scale (EPDS) Hamilton Depression Rating Scale (HAMD) Spielberger's State Anxiety Inventory (STAI)	IgA, but not IgM, responses directed against TRUCATs are higher in women with PMS compared with those without PMS. – No signification association between increased TRYCAT levels and perinatal depression, while we found even lowered anthranilic acid levels in perinatal depression. – No significant effect on IgA or IgM
Lizewska et al. (19)	Profile maternal plasma metabolome in spontaneous preterm birth.	group I—preterm 24 and 37 weeks (*n* = 57), group Ia—patients subdivided from group I, (between 24 and 37 weeks) within 7days after diagnosis (*n* = 37), group II—patients with symptoms of threatening preterm labor between 23 and 37 weeks of pregnancy who gave birth at term (*n* = 49), and group III patients who had blood collected during natural child-birth at term after normal pregnancy, between 38 and 41weeks of pregnancy (*n* = 25).	Retrospective case-control	Peripheral blood sample	Group I compared to group II, there was significantly different metabolites that belong mainly to fatty acids. Only one amino acid—tryptophan and one hormone metabolite pregnenolone sulfate were significantly different between these two groups (*p* < .05).
El-Heis et al. (20)	Examine the relation of maternal serum concentrations of nicotinamide and related tryptophan metabolites to the risk of atopic eczema in the offspring.	*n* = 497 mother-infant dyads	Cross-sectional	Maternal serum levels of kynurenine, kynurenic acid, anthranilic acid, tryptophan, nicotinamide, and N1-methylnicotinamide were measured in late pregnancy.	No association with offspring atopic eczema at age 6 months. However, univariate analyses of maternal and infant characteristics showed that higher maternal serum concentrations of nicotinamide and anthranilic acid were associated with lower odds ratios of atopic eczema at age 12 months No significant associations late pregnancy serum levels of tryptophan, kynurenine, kynurenic acid or N1-methylnicotinamide.Concentrations of nicotinamide and anthranilic acid showed a significant correlation (*r* = 0.218, *p* < 0.001
Lee et al. (21)	plasma biomarkers to predict the likelihood of adverse pregnancy outcomes (APO) in pregnant persons with SLE.	*n* = 33 pregnant women with SLE *n* = 10 normal pregnant controls, matched for gestational age.	Cross-sectional	Metabolomic studies of midtrimester maternal plasma.	SLE patients with severe APO reduced amino acids (e.g., methionine, tryptophan, and valine). An increase in lipophilic compounds, including saturated fatty acid (palmitic acid), polyunsaturated fatty acids (oleic acid, linoleic acid, and arachidonic acid), and branched fatty acid esters of hydroxy fatty acids (FAHFAs)., tryptophan, a biomarker constituent, showed a significantly lower level even after multiple comparison corrections (FDR, q-value ≥0.001).
Magnus et al. (22)	Examine the associations of maternal plasma total neopterin levels and KTRs in mid-pregnancy with asthma at age seven years.	*n* = 2,883 mother-child dyads	Longitudinal cohort	Mother:blood samples (collected around gestational week 18); from this, neopterin, kynurenine, and tryptophan levels were measured. Child: Asthma as defined by maternal report or recently (within the past 12 months) dispensed prescription asthma medication.	The mother's KTR was positively associated with parity, pre-pregnancy body mass index, and creatinine levels. No significant associations with asthma at 7 years of age
Shah et al. (23).	*in vitro* functional responses to common recall antigens, profile the changes in markers of peripheral blood T cell activation and maturation associated with pregnancy, and the *in vitro* expression of PIBF on lymphocytes in response to progesterone.	ELISA assays: *n* = 15 healthy volunteers *n* = 10 pregnant mothers. Peripheral blood flow cytometry: *n* = 10 healthy pregnant women in 2nd trimester *n* = 10 healthy pregnant women in 3rd trimester *n* = 9 healthy non-pregnant controls.	Longitudinal	Peripheral blood ELISAs Flow cytometry	Plasma neopterin concentration positively correlated with greater proportions of CD86 expressing mDC (*p* = 0.0320) and pDC (*p* = 0.0225). KTR positively correlated mDC (*p* = 0.0001) and CD86 expression on mDC (*p* = 0.0163).
Sikorski et al. (24)	Identify serum metabolomic signatures associated with gestational diabetes mellitus (GDM), and to examine if ethnic-specific differences exist between South Asian and white European women.	*n* = 600 GDM *n* = 133, No GDM *n* = 457	Prospective cohort study with nested case-control analysis	63 fasting serum metabolites were measured using multisegmented injection-capillary electrophoresis-mass spectrometry	Elevated circulating concentrations of glutamic acid, propionylcarnitine,tryptophan (*p* = .027), arginine, 2-hydroxybutyricacid,3-hydroxybutyricacid, and 3-methyl-2-oxovalericacidwere associated with higher odds of GDM, while higherglutamine, ornithine, oxoproline, cystine, glycine with lowerodds of GDM. Per SD increase in glucose concentration, the odds of GDM increased (OR = 2.07, 95% CI: 1.58 to2.71)

## 3 Results

The results of our review uncovered an array of study outcomes involving TRP and its metabolites from which several themes emerged, including TRP and TRP metabolites as potential biomarkers of psychological and physiologic outcomes in pregnancy, labor outcomes, pregnancy-specific complications, and non-pregnancy-related comorbidities, which can affect pregnancy outcomes.

### 3.1 Tryptophan and tryptophan metabolites as markers of maternal postpartum depression and anxiety

The most common theme among the studies included in this review was the involvement of TRP and its metabolites in maternal psychological symptoms. Of the 16 studies included, six focused on at least one psychological outcome, including perinatal depression (7, 11, 14, 15, 18) and anxiety (11, 16). One study evaluated anxiety in the setting of irritable bowel syndrome (IBS) (17).

Duan et al. (14) found that increased postpartum depressive symptoms (PDS) were associated with increased IDO activity (*n* = 725, *p* < .05). Additionally, single nucleotide polymorphisms (SNPs) of the IDO1 gene ex. rs10108662 were significantly related to PDS (*p* < .05). The authors did not find a significant difference in TRP levels at the end of term and postpartum day 1 in women with vs. without PDS. However, by postpartum day 3, PDS patients had lower TRP levels than those without PDS (*p* < .001). Additionally, KYN was higher in PDS, compared to those without PDS, at the end of term (*p* = .045) and postpartum days 1 and 3. The KTR in patients with PDS was also significantly higher than in those without PDS at the end of term (*p* = .01), postpartum day 1 (*p* = .02), and postpartum day 3 (*p* = .004), indicating increased IDO enzymatic activity in patients with PDS (14).

Roomruangwong et al. (18) evaluated the responses of serum IgM and IgA to TRP and 9 TRP catabolites (TRYCATs) in relation to perinatal depression and anxiety symptoms, among other variables. They found lowered serum IgA responses to anthranilic acid (which is a TRYCAT) levels in women (*n* = 126) with perinatal depressive symptoms. However, TRP and the other 8 TRYCATs had no significant IgM/IgA responses associated with perinatal depression or anxiety symptoms, indicating that TRYCAT pathway activation in the mucosa was not significantly associated with perinatal depression/anxiety symptoms (18).

Sha et al. (15) constructed prediction models, composed of 15 biomarkers including TRP and several of its metabolites, which could strongly predict depression, defined as an Edinburgh Postnatal Depression Scale (EPDS) score of >/=13. Specifically, they found that higher levels of the KYN pathway metabolite quinolinic acid in the third trimester were significantly associated with increased depression severity via EPDS score (*n* = 114, *p* = .04) and increased risk of scoring greater than 13 on the EPDS, indicating severe symptoms (41% OR increase per median absolute deviation, 95% CI: 1.8, 96.6; *p* = 0.02). Additionally, quinolinic acid and KYN, when measured in the second trimester, were strong individual predictors of depression severity and increased risk of severe depression symptoms occurring in the third trimester (15). In a 2015 study, not in pregnant women, KYN levels are notably higher in individuals who have attempted suicide and suffer from depression than in those with depression who have not attempted suicide, indicating a potential biomarker for suicidality in depressed patients (25). This elevation suggests that the activation of the kynurenine pathway could help differentiate suicidal from non-suicidal patients with depression (25). Additionally, the severity of depressive and suicidal symptoms in these patients varied directly with cytokine levels and inversely with KYNA levels, reinforcing the observation that reduced KYNA is associated with depression, yet may remain constant in non-suicidal depression cases (25).

Teshigawara et al. (7) reported that the post-partum depression group had higher levels of KYN and KYNA during pregnancy compared to those of the non-depressed group. Participants from the non-depressed group had higher postpartum levels of TRP, KYN, 3-hydroxyanthranilic acid (3-HA), and KYNA than during pregnancy. However, the authors found no significant difference in plasma levels of TRYCATs between the temporary gestational depressive group, continuous depressive group, and the non-depressive group. The postpartum to pregnancy plasma KYN ratio was significantly lower in the postpartum depression group compared to the non-depressed group (*n* = 132, *p* < .01). The KTR and KYNA: KYN ratios were higher during pregnancy for the postpartum depression group than in the non-depressive group (7).

Nazzari et al. (11) reported that higher scores on the Edinburgh Perinatal/Postnatal Depression Scale (EPDS) in late pregnancy were associated with lower prenatal KYN levels, and vice versa. However, no relationships between the prenatal levels of TRP or the KTR were found until after one year post-partum, where there was a significant interaction between IL-6, TRP, and time on EPDS scores. Observing the effect of prenatal TRP levels and IL-6 on EPDS scores during pregnancy, lower prenatal TRP and higher IL-6 levels had a positive association with depressive symptoms in late pregnancy (*n* = 110, *p* = .04). However, Nazzari et al. did not find that TRP, KYN, or the KTR had a significant effect on State Trait Anxiety Inventory (STAI) scores in late pregnancy or thereafter.

Keane et al. (17) reported that at 15 weeks gestation, the KTR in the healthy cohort indicated a positive correlation with STAI scores (*n* = 104). In the IBS cohort, decreased levels of TRP were found in moderate (*p* = .044) and low-scoring groups (*p* = .013). The low-scoring group had the highest concentration of KYN, compared to the moderate (*p* = .017) and high-scoring groups (*p* = .014). Lower levels of IL-8 (*p* = .001) and TRP (*p* = .008) were found at 20 weeks gestation compared to 15 weeks gestation in healthy and IBS mothers. A significant drop in TRP and KYN occurred at 20 weeks in the IBS cohort (*p* = .031, *p* = .028).

**Figure 3:**
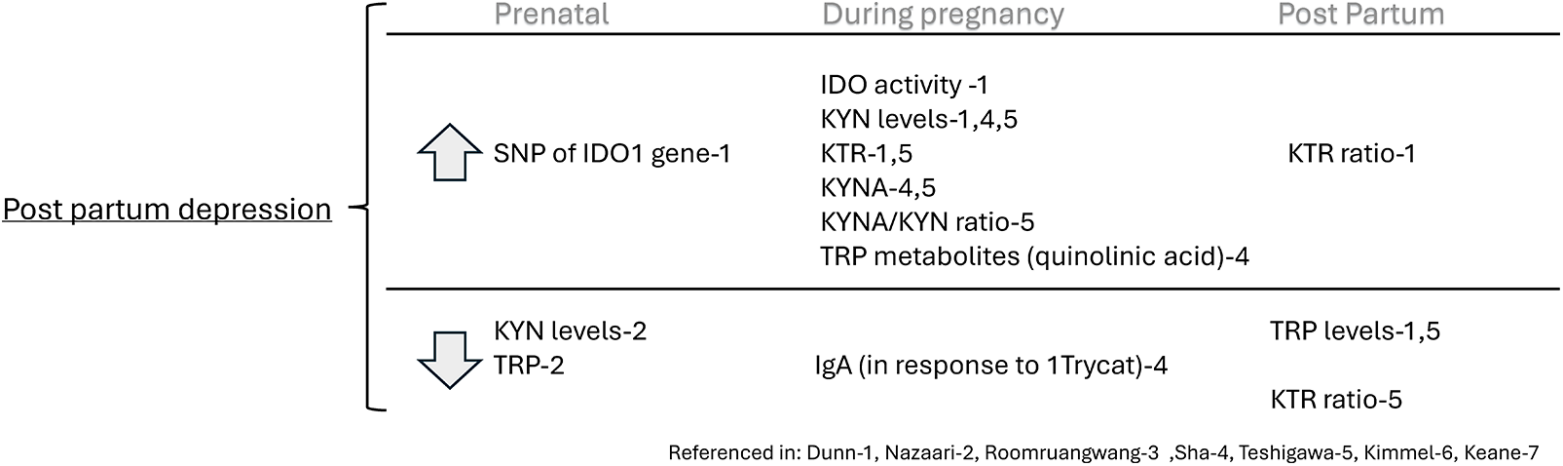
Tryptophan and tryptophan metabolites as markers of perinatal depression.

Together these studies reveal that TRP levels are decreased in women with depressive symptoms before and after pregnancy but are not different in women with depression or anxiety during pregnancy, though the TRP-KYN pathway is disturbed during pregnancy at several different points including KYN and KYNA levels and the KTR and KYNA/KYN ratios in women with depressive symptoms or those that develop postpartum depression. See Figure 3.

### 3.2 Tryptophan and tryptophan metabolites as markers of prolonged and preterm labor

TRP and its metabolites may be related to adverse pregnancy outcomes and preterm delivery. For instance, when maternal BMI and age are controlled, there was a significant difference in TRP metabolism between the labor dystocia cases and control groups (*p* = .021) in which TRP levels in the circulating serum of women in the control group were elevated compared to the women in the labor dystocia group (*p* = .03–.004) (13).

**Figure 4:**
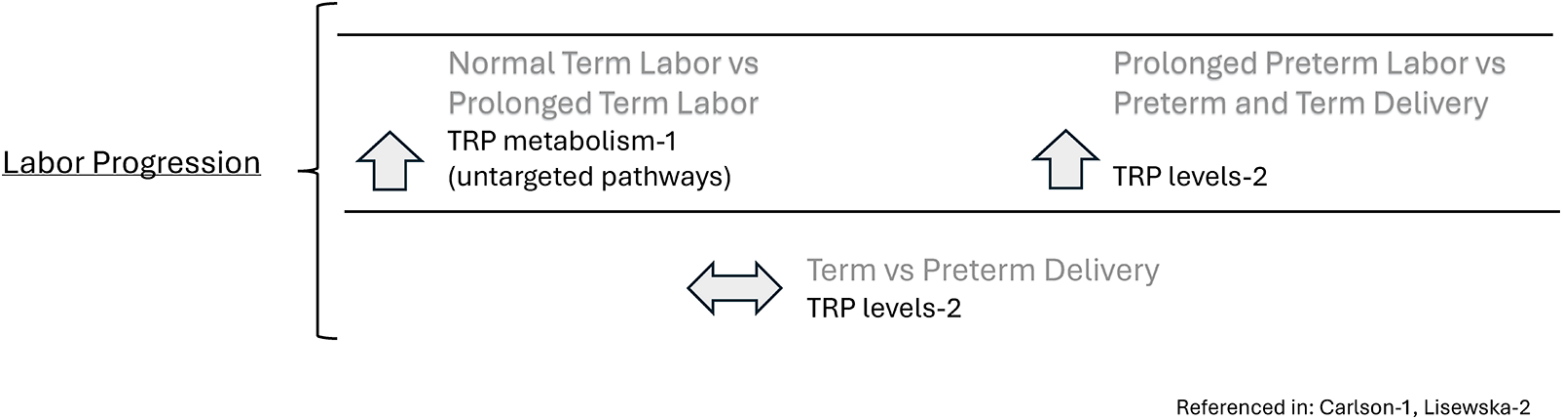
Tryptophan and tryptophan metabolites as markers of labor progression.

Additionally, when considering levels of TRP and its metabolites in spontaneous preterm births, TRP differed significantly in a metabolomics study between the preterm labor group and the threatened preterm labor group, who delivered to term (19). For instance, TRP was significantly lower (*n* = 86, *p* < .05) in the preterm delivery group compared to the threatened preterm delivery group (19). Intriguingly, TRP levels were significantly higher in the threatened preterm delivery group compared to the normal term delivery group (*n* = 25 *p* < .0001) though this sample size was small and may not be representative of all pregnancies, and the samples were obtained at 31 weeks vs. 39 weeks so may reflect changes that occur during the course of gestation (19). There were no significant differences in TRP levels between term and preterm deliveries (19). See Figure 4.

### 3.3 Tryptophan and tryptophan metabolites as markers of physiologic outcomes in pregnancy

#### 3.3.1 Gestational hypertension and pre-eclampsia

**Figure 5:**
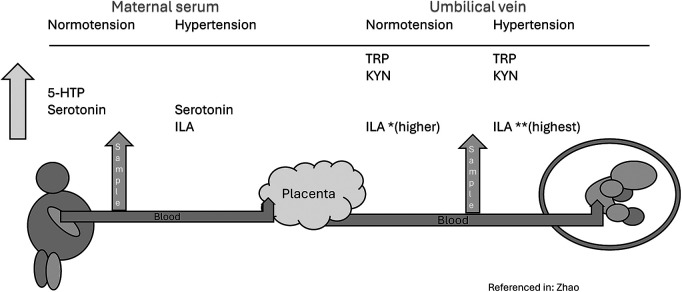
Tryptophan and tryptophan metabolites in pre-eclampsia.

TRP metabolite levels may also play a role in gestational complications such as pre-eclampsia. TRP and KYN are higher in the umbilical vein vs. maternal serum in both the normotensive and hypertensive groups, while the metabolite, 5-HTP, is higher in maternal sera only in the normotensive group (12). Serotonin is higher in maternal serum in both hypertensive and normotensive groups compared to the umbilical vein samples, while the metabolite, Indole-3-lactic acid (ILA), was increasingly higher in the preeclamptic group compared to the normotensive group in both the maternal serum and the umbilical vein (*p* < .05) indicating that the concentrations of TRP and its metabolites are altered in the setting of preeclampsia, resulting in elevated levels of indole-3-lactic acid (ILA). See Figure 5.

#### 3.3.2 Metabolic changes in pregnancy

Two articles evaluated TRP's role in metabolic changes during pregnancy. Magnus et al. (22) assessed the KTR during pregnancy in relation to childhood asthma (22). Magnus et al. found that KTR was positively associated with pre-pregnancy BMI. Pregnant women with overweight or obese BMIs showed a significantly higher amount of neopterin and KTR during gestation than those with underweight or normal weight BMIs. Underweight and normal weight BMI pregnancies reported a median of 6.74 and 7.01 nmol/L of neopterin levels and a median of 18.11 and 18.30 nmol/μmol of KTR, respectively; overweight and obese BMI pregnancies reported a median of 7.27 and 8.19 nmol/L of neopterin levels and a median of 18.83 and 20.09 nmol/μmol of KTR levels, respectively (22). Sikorski et al. (24) evaluated metabolic signatures in gestational diabetes (GDM) and found higher TRP levels associated with GDM despite pre-pregnancy BMI. BMI was also found to correlate positively with TRP metabolism during pregnancy, though Asian women had higher incidence of GDM despite having lower pre-pregnancy BMI than their European counterparts (22). Together these studies indicate that TRP and TRP metabolites are associated with metabolism and alterations in metabolism that occur during pregnancy or that may be associated with differences in ethnicity.

#### 3.3.3 Tryptophan and the immune System's adaptation to gestation

In addition to its role in metabolic pathways during pregnancy, TRP has been studied in maternal and fetal immune-related outcomes. Silvano and colleagues reported that maternal plasma KTR was not significantly associated with placental expression of IDO1or TDO in the in healthy term pregnancies tested just after delivery (5).

Shah et al. (23) found a positive correlation between maternal serum KYN and the proportion of mDC (*p* = .0001) and CD86 expression on mDC (*p* = .016) and a positive correlation between maternal plasma neopterin concentration and the proportion of CD86 expressing mDC (*p* = .03) and pDC (*p* = .02) and CD86 MFI on mDC (*p* = .012). These correlations were more robust in the third trimester. Changes in KTR were associated with reduced TRP concentration throughout pregnancy (*p* = .025) (23).

While examining adverse pregnancy outcomes in women with systemic lupus erythematosus (SLE), Lee et al. (21) noted a 0.65-fold decrease in plasma concentration of TRP (*p* = .001) between women with SLE who had adverse pregnancy outcomes (preterm delivery, preeclampsia, small for gestational age, intrauterine fetal death, etc.) compared to women with SLE who had normal pregnancy outcomes.

Further evaluating the association between TRP and immunological outcomes in pregnancy, two studies examined maternal TRP's impact on long-term immune-related outcomes such as eczema and asthma in infants. Magnus et al. (22) did not find any significant associations between asthma development by age seven and maternal KTRs during pregnancy, and El-Heis et al. (20) found no significant associations between third-trimester maternal serum levels of TRP, KYN, or KYNA and the development of infant atopic eczema at 6 or 12 months of age. However, there was a significantly lower risk of developing infant atopic eczema at 12 months of age associated with higher maternal serum concentrations of nicotinamide (*p* = .013) and anthranilic acid (*p* = .003) after controlling for relevant maternal and confounding variables (20). Together these findings indicate that TRP and its metabolites are markers of physiologic and pathophysiologic immune dysregulation in pregnancy, which may indicate adverse labor and pregnancy outcomes, but do not necessarily predict long-term immunologic outcomes without associated conditions of increased inflammation such as obesity or autoimmunity. See Table 2.

**Table 2:** Tryptophan and tryptophan metabolites and immune interactions in pregnancy.

Study	Variable 1	Variable 2	Association	When tested
Silvano	KTR	Placental IDO	ns	At delivery
		Placental TDO	ns	At delivery
Shah	KYN	Myeloid DC (mDC) (tolerogenic)	+ correlation	3rd trimester
		CD86 (costimulatory signal for T-cell activation) on mDC	+ correlation	3rd trimester
	Changes in KTR	Decreased TRP		Throughout pregnancy
Lee	Preterm, PIH, SGA, fetal mortality	Decreased TRP	Inverse correlation to TRP	In women with SLE
Magnus	Asthma by 7	KTR	ns	
El Heis	Eczema 6–12 mos.	TRP, Kynurenine, KA	Ns, ns, ns	3rd trimester
	Eczema 12 mos. (low)	Nicotinamide (vit B3), anthranilic acid (high)	Inverse correlation	
Roomruangwong	TRP	IgM/IgA	ns	PPD or Anxiety
	IgA response to	Trycat anthranilic acid decreased	Inverse correlation	PPD
Nazzari	TRP low and IL6 high	Depression	+ correlation	
		Anxiety	ns	
Keane	TRP low and IL8 lower at 20 weeks vs. 15 weeks in all	TRP, IL8 drops at 20 weeks in IBS only		Healthy and IBS mothers
Magnus	Neopterin and KTR higher in overweight or obese	Neopterin and KTR decreased in normal or underweight	Overweight and obese vs. normal and underweight	

## 4 Discussion

We conducted this review to synthesize current knowledge on maternal plasma TRP and its metabolites to understand if TRP or its metabolites may be useful targets as biomarkers of adverse pregnancy outcomes or perhaps as targets to improve pregnancy outcomes. We found that TRP and its metabolites may be markers of maternal depression and anxiety, labor dystocia, gestational hypertension, and as a signal of dysregulated immune function.

Certain risk factors for anxiety and depression are well known, such as a history of mental illness especially in a primigravida pregnancy. To date, there are no physiologic screening tests utilizing biomarkers to screen for anxiety and depression in pregnancy. It is unclear why psychological disorders occur in some pregnant women and not others (26). TRP and its metabolites pose promising insights into psychological pathophysiology during pregnancy, but research is needed to elucidate when maladaptive changes in the TRP pathway are most useful for identifying women at risk for perinatal depression and anxiety.

TRP may be a promising biomarker for perinatal depression due to its role as a precursor in serotonin synthesis, a key neurotransmitter involved in mood regulation. The variability in TRP levels linked to different outcomes in pregnancy, such as preterm birth, suggests that changes in the TRP pathway could influence mood disorders (7). Understanding this mechanism could lead to early identification of depression risk in pregnant women through TRP level monitoring. With this knowledge, early interventions, such as dietary modifications to increase TRP intake or medical therapies to regulate serotonin levels, could be implemented to prevent or mitigate perinatal depression.

The role of TRP and its metabolites is less evident in perinatal anxiety, with relatively few studies showing mixed results. The role of TRP and its metabolites in perinatal anxiety is less specific compared to their established link with depression. While TRP is a precursor for serotonin, a neurotransmitter tied to mood and anxiety, its link to anxiety, specifically during pregnancy, shows mixed results. For instance, a study found that higher plasma TRP concentrations were associated with better sleep quality during pregnancy, especially among those with anxiety symptoms. However, no direct correlation with anxiety itself was observed (11). This suggests that while TRP may influence factors that contribute to mental well-being, and its role as a direct biomarker for anxiety during pregnancy is not firmly established.

Further research into the TRP metabolism pathway, particularly the conversion of TRP to KYN, may provide more insights. For example, enhanced metabolism of TRP into KYN due to increased inflammation has been linked with depressive but not anxiety symptoms across the perinatal and postpartum period in a low-risk sample (11). This indicates that while the TRP pathway's involvement in emotional regulation during pregnancy is supported, its effect on anxiety specifically requires further investigation.

If a reliable link were established, monitoring TRP levels could potentially allow for the early identification of women at risk for perinatal anxiety. Early detection could lead to proactive measures, including dietary interventions to ensure adequate TRP intake, which might help maintain mental well-being. Because the TRP pathway is dynamic throughout pregnancy, careful comparisons that rigorously control for the week of gestation in which the samples were drawn in addition to sampling of metabolites indicative of which pathway is activated are needed to help understand the characteristics that identify or classify anxiety and depression in pregnant or post-partum women. Until more definitive results are available, however, the use of TRP levels as a biomarker for perinatal anxiety remains a topic for future research rather than a current clinical practice.

Labor-related conditions and outcomes, such as labor dystocia and preterm delivery, are notably associated with TRP and its metabolites. Research by Carlson et al. (13) indicates that women with labor dystocia have lower TRP levels in maternal serum than controls. Lizewska et al. (19) found a similar pattern of low maternal TRP levels in preterm births. These findings collectively suggest an association between reduced TRP levels and adverse birth outcomes, including labor dystocia and preterm birth. The mechanism behind this pattern may involve the role of TRP as a precursor for serotonin, a neurotransmitter that influences mood, uterine contractility, and the stress response. Lower levels of TRP could result in reduced serotonin synthesis, which might affect the uterine environment and the ability to sustain a full-term pregnancy or undergo a successful labor process.

Furthermore, TRP metabolism via the KYN pathway involves immune modulation, which is critical during pregnancy. The enzymes in this pathway are highly expressed in the placenta and play a pivotal role in managing the maternal-fetal immune response. A deeper understanding of these immune mechanisms is crucial for clarifying the mechanisms that contribute to the pathogenesis of pregnancy complications. Throughout pregnancy, there is a shift towards an active immunological tolerance supported by a network of hormones, cytokines, and various immune cells. This shift ensures both the facilitation of fetal development and the preservation of the mother's ability to counter infections. The presence of regulatory T cells and associated immune mediators in the uterus aids placental development and function while simultaneously restricting the activity of effector T cells, thus protecting the placenta from the maternal immune system's potentially harmful elements. Understanding these immunometabolic interactions could lead to the development of preventive interventions for pregnancy-related disorders. The uterus is not an immune-privileged site but rather one of active immune tolerance and could guide novel therapies for complications like pre-eclampsia by potentially using endogenous vasorelaxants such as KYN to treat hypertension. Such insights highlight the potential for targeting immune pathways as a path to improved pregnancy outcomes.

Emerging research underscores the role of TRP degradation in modulating maternal immune function, with implications for fetal tolerance. The IDO pathway, which involves TRP degradation, is crucial for maintaining pregnancy by regulating maternal immune responses and preventing fetal rejection (27). Lee's 2019 study draws a parallel between heightened TRP degradation and autoimmune conditions, observing that systemic lupus erythematosus (SLE) patients with adverse pregnancy outcomes (APO) often suffer from exacerbated disease activity, potentially linking disrupted TRP metabolism to immune dysregulation. Moreover, the perturbation of TRP pathways, evidenced by altered KYN levels, has been connected to T cell dysfunction and chronic fatigue in SLE, suggesting a broad immunological impact of TRP metabolism during pregnancy (21).

However, the study did reveal that the metabolite ILA (Indole 3 lactic acid) was significantly higher in the pre-eclamptic group compared to those with normotensive pregnancies, though a recent study by Zhao et al. (12) found no differences in pre-eclampsia patients vs. healthy controls. ILA may, however, have potential significance in the development of pre-eclampsia. ILA, derived from TRP metabolism by gut microbiota, has been identified for its anti-inflammatory properties, particularly in the immature intestine. This could be significant in the development of pre-eclampsia, a condition marked by inflammation. ILA's potential to modulate inflammatory responses in pregnancy may influence the risk of developing pre-eclampsia, suggesting a need for further exploration of its therapeutic potential (28). Currently, proteinuria and hypertension are the primary indicators used to identify pre-eclampsia. Still, ILA could serve as a biological predictor of the condition if levels are significantly higher before onset. While the study measured ILA levels after pre-eclampsia diagnosis, future research could explore ILA as a predictor of pre-eclampsia occurrence by measuring levels before diagnosis, thus providing valuable screening information for clinicians.

TRP plays a pivotal role in maternal body mass index (BMI) regulation during pregnancy and is implicated in developing gestational diabetes mellitus (GDM). Elevated TRP levels can trigger heightened activity in the TRP-KYN pathway, a phenomenon more frequently observed in pregnant individuals with pre-existing GDM (24). This suggests that TRP may contribute to BMI changes during pregnancy and has potential implications for the development of GDM. Further investigation is necessary to understand the mechanisms and clinical significance of this relationship fully.

These findings collectively highlight the diverse roles of TRP and its metabolites in perinatal mental health and pregnancy outcomes, underscoring the complexity of their influence. While TRP shows promise as a biomarker for perinatal depression and may have implications for other conditions, it is essential to acknowledge certain limitations in the existing research surrounding clinical utility and specific involvement in maternal health outcomes.

### 4.1 Limitations

The body of literature regarding TRP as a biomarker of pregnancy-related outcomes contains methodological differences between studies that challenge our ability to make reliable conclusions at this time but suggest many avenues for further investigation. A majority of the studies have small sample sizes, which hinder generalization (5, 7, 12–17, 20, 21, 23). The retrospective design of some studies and the inclusion of specific population subsets or stages of pregnancy in others limits the ability to infer the effects of TRP on metabolism and pregnancy outcomes (11, 13, 15, 17, 20, 23). As noted by Keane et al. (17) and Nazzari et al. (11), a dependency on self-reported data may introduce bias, underscoring the need for triangulation with objective data. Future research could benefit from more inclusive sampling strategies and longitudinal designs that track changes in TRP metabolism throughout pregnancy (11, 19, 24). Moreover, it is essential to use precise diagnostic tools for psychological conditions such as depression to better understand how TRP levels relate to perinatal depression outcomes (18).

Studying TRP during pregnancy could enhance our understanding of its multifaceted role across varied maternal experiences (7). Furthermore, conducting research across multiple centers would enable a detailed comparison of how TRP functions as a biomarker within different populations and healthcare environments, thus enriching our insight into its biological and psychological influence (21). Critically, our understanding of TRP and its metabolites as measured in maternal plasma would be enhanced by rigorous control of both the timing of sampling, and the acquisition of downstream indicators of TRP metabolic pathway changes. Understanding the changes in plasma free TRP concentration, which depends on its binding to albumin and may be modified by non-esterified fatty acids levels in pregnancy is also of critical importance in understanding mechanisms of TRP metabolism that are altered in maternal and fetal pathologic conditions (8, 29).

## 5 Conclusion

In conclusion, this integrative review underscores the potential of TRP as a multifaceted biomarker for predicting a range of pregnancy-related outcomes. The evidence compiled highlights TRP's involvement in psychological conditions such as perinatal mood disorders, its metabolic role amidst the physiological changes during pregnancy, and its function within the immune system's adaptation to gestation (5, 24, 28). Moreover, evidence suggests a link between TRP metabolism and immune system changes during pregnancy, which could influence both maternal and neonatal health (7, 23).

While the promise of TRP is clear, further longitudinal and mechanistic studies are needed to fully elucidate its biomarker capabilities across diverse populations and complex conditions. This knowledge could pave the way for improved screening, prevention, and treatment strategies, contributing to better pregnancy-related outcomes. Collectively, the multifunctionality of TRP as a biomarker signals its potential utility in monitoring and managing pregnancy complications. Future research should continue exploring TRP's complex biological interactions within the gravid state, ensuring its promise translates into tangible clinical benefits.
